# Emotion regulation training in the treatment of obesity in young adolescents: protocol for a randomized controlled trial

**DOI:** 10.1186/s13063-019-4020-1

**Published:** 2020-02-10

**Authors:** Taaike Debeuf, Sandra Verbeken, Elisa Boelens, Brenda Volkaert, Eva Van Malderen, Nathalie Michels, Caroline Braet

**Affiliations:** Department of Developmental, Personality and Social Psychology, Ghent University, Ghent, Belgium

**Keywords:** Obesity, Emotion regulation training, Youngsters, Stress

## Abstract

**Background:**

The prevalence rates of childhood obesity are increasing. The current multidisciplinary treatments for (childhood) obesity are effective but only moderately and in the short term. A possible explanation for the onset and maintenance of childhood obesity is that it reflects a maladaptive mechanism for regulating high levels of stress and emotions. Therefore, the current RCT study aims to test the effectiveness of adding an emotion regulation training to care as usual (multidisciplinary obesity treatment) in young inpatients (10–14) involved in an obesity treatment program compared to care as usual alone.

The research model for this RCT study states that when high levels of stress are regulated in a maladaptive way, this can contribute to the development of obesity.

**Methods:**

The current study will recruit 140 youngsters (10–14 years) who are involved in an inpatient multidisciplinary obesity treatment (MOT) program. After giving consent to participate in the study, youngsters will be randomly assigned, during consecutive waves, to one of two conditions: care as usual (receiving MOT) or intervention (receiving MOT in addition to emotion regulation training). The training itself consists of 12 weekly sessions, followed by a booster session after 3 and 5 months. The participants will be tested pretraining, post-training, and at 6 months’ follow-up. We hypothesize that, compared to the control condition, youngsters in the intervention condition will (1) use more adaptive emotion regulation strategies and (2) report less emotional eating, both primary outcome measures. Moreover, on the level of secondary outcome measures, we hypothesize that youngsters in the intervention condition, compared with the control condition, will (3) report better sleep quality, (4) undergo improved weight loss and weight loss maintenance, and (5) experience better long-term (6-months) psychological well-being.

**Discussion:**

This study will add to both the scientific and clinical literature on the role of emotion regulation in the development and maintenance of different psychopathologies, as emotion regulation is a transdiagnostic factor.

**Trial registration:**

The RCT study protocol is registered at ISRCTN Registry, with study ID “ISRCTN 83822934.” Registered on 13 December 2017.

## Background

### Childhood obesity: growing prevalence, multiple consequences, and modest treatment success

Recent prevalence rates collected by the World Health Organization [150] revealed that, worldwide, 18% of the school-aged children and adolescents (5–19 years old) are diagnosed with overweight and 8% with obesity. These prevalence rates have tripled since 1975, demonstrating the growing problem of pediatric overweight and obesity [150]. This causes a great concern for both the individual and the community, as obesity has high medical and psychosocial consequences, both in the short term and long term [153].

The well-researched and currently golden standard treatment for childhood obesity is Multidisciplinary Obesity Treatment (MOT). The overall goal of the treatment is to improve the distorted balance between the energy intake and energy expenditure by changing the diet and increasing the physical activity [29, 106]. MOT incorporates cognitive behavioral therapy with active involvement of the parents to support behavior change and enhance the psychosocial well-being [29, 106]. Positive effects of MOT have already been found on weight (body mass index, BMI − 0.5 SD), physical fitness, and psychosocial well-being [104, 113]. However, the long-term effects of MOT are still limited, as weight-loss maintenance seems very hard to attain [4, 29, 96, 103, 151].

In sum, the high prevalence rates of childhood obesity; the medical, psychological, and economic consequences; and the poor long-term treatment effects emphasize the need to study in depth all mechanisms leading to childhood obesity. Herein, a psychological perspective recently has gained attention. An increasing number of studies point to “stress” as an important factor in the development and maintenance of childhood obesity [8, 74, 79, 110]. For example, families with an obese child experience more stress [102], and daily hassles seem to have a significant impact on children’s eating behavior [44, 110]. However, although the contributing role of stress in obesity is well studied, treatment options have not been developed in response. Therefore, the present study will bridge this gap by evaluating the role of treatment in an RCT study.

### Stress conceptualization

Stress occurs when a person perceives (goal-related) danger, attaches personal significance to the situation for his or her well-being, and the available coping resources of the person fail to deal with the situation [88]. Next to the cognitive and emotional component, stress also contains a physiological component [88]. The latter can be seen as a related but different indicator of a stress experience [143], reflecting the physiological arousal, which prepares the person to cope with the stressor by fighting or fleeing, and the restoration of homeostasis. Two main physiological stress systems have been identified [36]. The first stress system is the hypothalamic-pituitary-adrenal axis with cortisol as the end product. The second stress system is the autonomic nervous system with the catecholamines, adrenaline, and noradrenaline as end products, for which heart rate variability (HRV) is used as a noninvasive biomarker to indirectly measure cardiac parasympathetic and sympathetic activity [91].

**Fig. 1:**
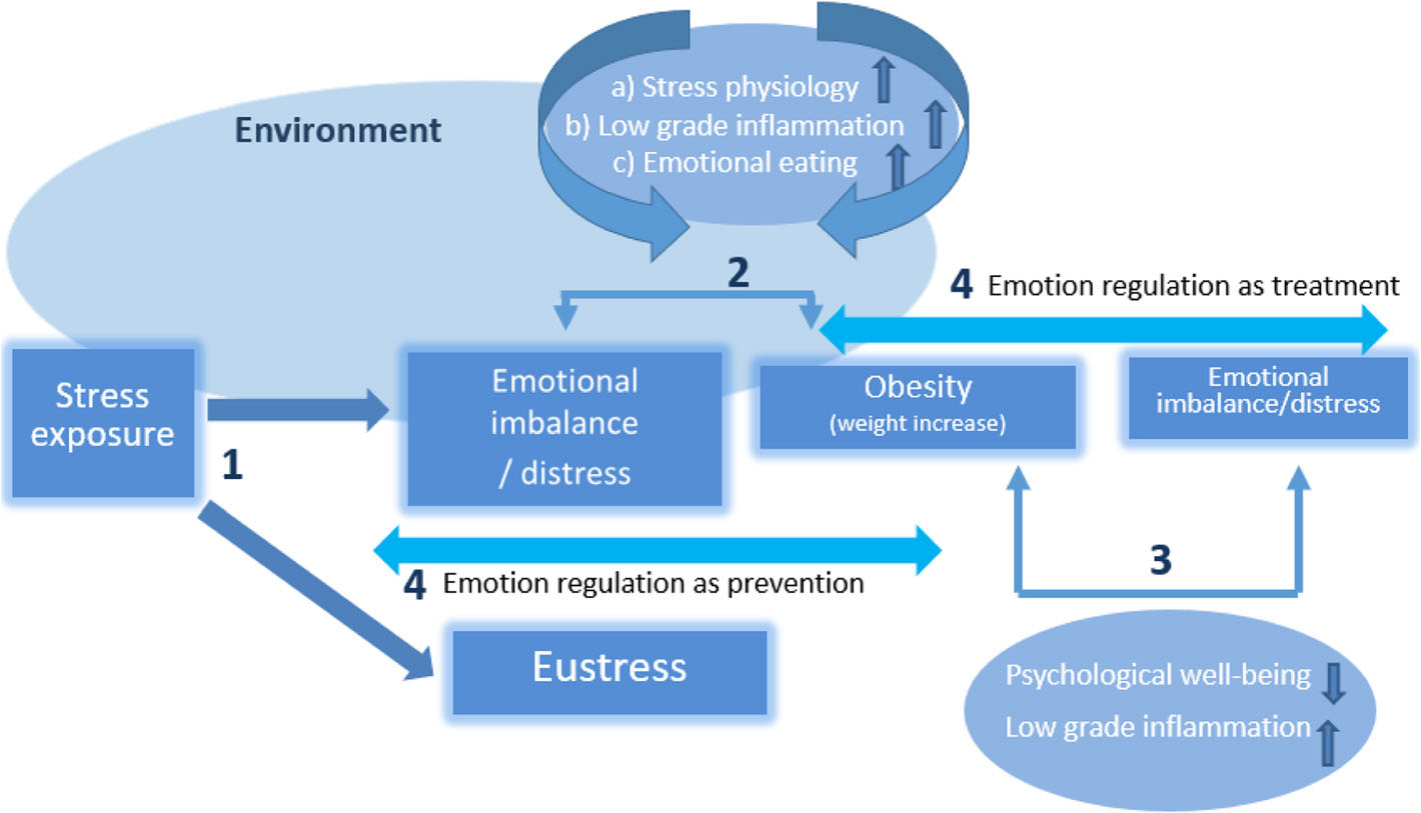
Emotion regulation as underlying mechanism

When stress is successfully managed, the “emotional homeostasis” will return, and in the long run, the emotional stability will not be fundamentally affected (see Fig. 1: eustress). Adversely, when the stress is not successfully managed, enhanced arousal and negative affects occur like chronic experiences of tension, danger, frustration, or rejection (see Fig. 1: emotional distress) [37, 70, 86]. Emotional distress is associated with psychopathology [39, 83, 97], medical diseases [125, 127, 129], and obesity [8, 42, 58, 79, 101, 123].

### A framework for the stress–obesity relationship

For a thorough understanding on how emotional distress may be associated with weight gain and obesity, three processes need to be considered (see Fig. 1). First, when stress is not successfully managed, chronic activation of the physiological stress tonus entails, which results in an elevated cortisol production. Chronic cortisol hypersecretion is directly associated with the accumulation of fat by stimulation of the fat cell growth and dysregulation of the lipolysis. Indirectly, the elevated cortisol level increases the appetite, calorie consumption, and consequently the adiposity [21, 36, 116]. Second, emotional distress triggers a low-grade inflammatory process, which occurs directly by elevating the cytokine production and indirectly by its association with less sleep, imbalanced diet, and less physical activity that subsequently induce inflammation [71, 89]. This inflammation process may stimulate other hypothalamic pathways, resulting in increased food intake and finally obesity by resistance to the appetite-reducing factors leptin and insulin [31, 32]. The third mechanism concerns emotional or stress-induced eating, defined as “overeating in response to emotions and stress” [8, 28, 48]. When experiencing stress, people aim to neutralize the stress-related arousal by eating, even in the absence of hunger, as this gives a warm and good feeling in the short term. Especially eating comfort food (e.g., highly palatable food which is rich in sugar and fat) is known as reinforcing [90]. Moreover, it initiates an automatic learning process [48, 90, 110], facilitating emotional eating as the preferred behavior when feeling emotionally distressed. This is observed in both normal weight and overweight adults and children [24, 107, 142]. As emotional eating can foster eating beyond the saturation point, it can lead to an increased calorie intake and to overweight or obesity over time [55, 84].

Importantly, the relationship between emotional distress and obesity is not unidirectional but a vicious cycle can be assumed: obesity itself will lead to further emotional imbalance (e.g., physical and psychological well-being), which is hypothesized to further induce (a) enhanced cortisol, (b) low-grade inflammation, and (c) emotional eating (see Fig. 1) [3, 28, 92, 119, 134, 146, 149]. Furthermore, excessive body fat directly maintains the low-grade inflammation that may lead to higher stress sensitivity and a maintained emotional imbalance, as inflammatory cytokines from excessive body fat can affect the brain and its stress-related neuro-circuitry, neuroendocrine activity, and neurotransmitters [34, 75].

The above-mentioned drastic processes, resulting from unsuccessfully regulated stress, highlight the question as to why some people seem able to regulate their stress, while others are unable to do so. Although it might be that some experience more stressors [102], it is also plausible that some are less capable of regulating stress-related emotions, leading to chronic stress experiences. This brings us to the hypothesis that emotion regulation is playing a crucial role in the vicious cycle of obesity (Fig. 1).

### Emotion regulation: the missing link?

Emotion regulation (ER) refers to the actions by which individuals try to exert an influence on the emotions they have, when and how they experience and communicate them [67]. These actions implicate the use of several ER competences which refer both to ER abilities (e.g., demonstrating compassion) and strategies (e.g., cognitive reappraisal). ER is recently found to affect different psychological problems, such as depression, anxiety, chronic pain, and ADHD [9, 73, 109, 124], and is now recognized as a transdiagnostic factor playing a role in the etiology and maintenance of different kinds of psychological problems [64]. Based on the long-term effects on affect, behavior, and psychopathology, Aldao and colleagues [5, 6, 68] suggest a classification in adaptive ER strategies and maladaptive ER strategies. Especially for obesity, studies have found that the association between stress and weight gain is stronger when more maladaptive ER strategies are used, whereas ER seems to mediate the stress-eating relationship [8, 48, 144]. Interesting, recent studies also emphasize the need to research adaptive ER strategies, as these are protective factors [27]. Commonly, in the literature, three adaptive ER strategies, “cognitive reappraisal,” “acceptance,” and “problem solving” have been researched and are found to be associated with less psychopathology (2009). Moreover, the adaptive ER abilities “acceptance,” “support yourself,” and the strategy “analyze and reappraise” are found to be trainable with positive effects in young adolescents [147, 148]. However, next to learning these ER competencies, also the sequence of applying them when feeling stressed is important [95, 111]. Therefore, Berking and Whitley [18] combined all these theoretical insights on ER competencies into a conceptual theory and training model on ER in adults, called Affect Regulation Training (ART). The ART model is in accordance with the recent literature on ER flexibility, referring to the importance of momentary flexibility in the use of different ER abilities and ER strategies across different stressful contexts taken into account the personal goals [7, 23]. Interesting, ART, both as a stand-alone intervention and on top of care as usual, is shown effective in clinical and nonclinical adult populations [15, 17, 18, 60]. Currently, our research group is evaluating the ER sequence, based on Berking and Whitley [18], in a prevention program for children and adolescents in schools [147], but it has not yet been evaluated in the context of emotional eating in a sample of obese children. However, given the evidence for the mediating role and the trainability of ER, we assume that an ER training will have good effects also for obese children. Therefore, in the preparation phase of the current RCT study, the feasibility of learning ER abilities and adaptive ER strategies in a population of obese youngsters has been investigated and well evaluated (Boelens, Debeuf, Verbeken, Volkaert & Braet: A Two-Hour Emotion Regulation Workshop in Early Adolescents with Obesity: A Feasibility Study, in preparation).

### Goals and hypotheses

As in the presence of stress, flexible and adaptive ER is a requisite to achieve eustress and avoid the detrimental pathways to weight (re)gain and obesity, an ER training may empower the current MOT and foster long-term weight loss maintenance (see Fig. 1). The present study will therefore examine the effectiveness of a newly developed 12-week ER group training (one training session of 2 hours per week) on top of the MOT by means of a randomized controlled trial (RCT) in an inpatient treatment center for childhood obesity. The addition of an ER training to the MOT compared with MOT “as usual” is hypothesized to result in 1) an improvement in ER competencies (assessed by means of self-report questionnaires, idiosyncratic measures, and validated with physiological parameters during a lab task) (=primary outcome); 2) less emotional eating behavior (assessed by means of self-report questionnaires and in a stress paradigm); 3) better sleep quality (assessed by means of self-report questionnaires); 4) improved weight-loss and weight-loss maintenance (in terms of adjusted BMI and waist circumference) with associated change in metabolic parameters; and 5) better long-term (6-month) psychological well-being (assessed by means of self– and parent report questionnaires). The protocol was registered at ISRCTN Registry with study ID: ISRCTN 83822934.

## Methods/Design

### Participants

Participants will be recruited in an inpatient treatment center for obesity in Belgium, *Het Zeepreventorium*, between March 2018 and September 2020. During consecutive waves, recruitment takes place at intake (T0; 4 months before starting the inpatient treatment) and both the youngsters and one of the parents should participate in the study. Inclusion criteria of the study are (1) 10–14 years old at the start of the inpatient treatment; (2) primary obesity, with minimum 60% overweight at intake in the treatment center according to the overweight index of Cole, Bellizzi, Flegal, and Dietz [38]; and (3) language proficiency, with mastery of the dominant language.

### Procedure

At recruitment (T0), the youngsters meeting the inclusion criteria and at least one of their parents will both receive detailed information from the researcher and the psychologists of the inpatient treatment center. After receiving this information on the study procedure orally and by letter; the youngsters and at least one of their parents will fill out an active informed consent. To obtain the target number of participants, *N* = 140 (power analysis, see below), a 2 ½ year recruitment period, March 2018 until September 2020, is necessary. After giving informed consent, participants are randomly assigned (see Randomization) to one of the two conditions: intervention condition or control condition. In the intervention condition, the ER training sessions will be given by trainers, who were also involved as clinical psychologists in the development of the ER training.

The Ethics Committee of the Ghent University Hospital approved the study design, procedure, and data collection. The national laws and the Helsinki Declaration of 1964 will be applied in all data collection procedures.

### Study design

The study design concerns a two-arm randomized controlled trial, evaluating the effects of an emotion regulation training intervention versus a care-as-usual control group. Assessments in the two conditions are planned at four time points: after recruitment (T0), before the start of the intervention (T1), after the end of the intervention (T2), and at 6-months’ follow-up (T3). Primary outcome measures are emotion regulation and eating behavior, more specifically emotional eating. As secondary outcome measures, (1) weight change, (2) psychological well-being, and (3) sleep quality will be taken into account.

After giving informed consent (T0), participants and their parents will fill out questionnaires assessing the psychological well-being, ER capacities, and perceived stress of the child (parent- and self-report), which will be used for descriptive purposes or as covariates. Next, before the start of the intervention (T1), at the end of the intervention (T2), and at 6 months’ follow-up (T3), participants will fill out questionnaires on their psychological well-being, sleep quality, ER capacities, eating behavior, and perceived stress level, and they will participate in a stress paradigm. On top, at T1, the participants will also complete the Standard Progressive Matrices test to determine their overall cognitive ability. Parents of the participants will be asked again to fill out questionnaires on the psychological well-being and the eating behavior of their child at T2 and T3. Moreover, the participants in the intervention condition will be asked to fill out a diary three times a week on their stress level, affect, ER capacities, and emotional eating behavior between the ER training sessions. Filling out a diary enables a more momentary inspection on the relationship between the stress level, ER capacities, and emotional eating behavior in the naturalistic environment of the participants.

**Fig. 2:**
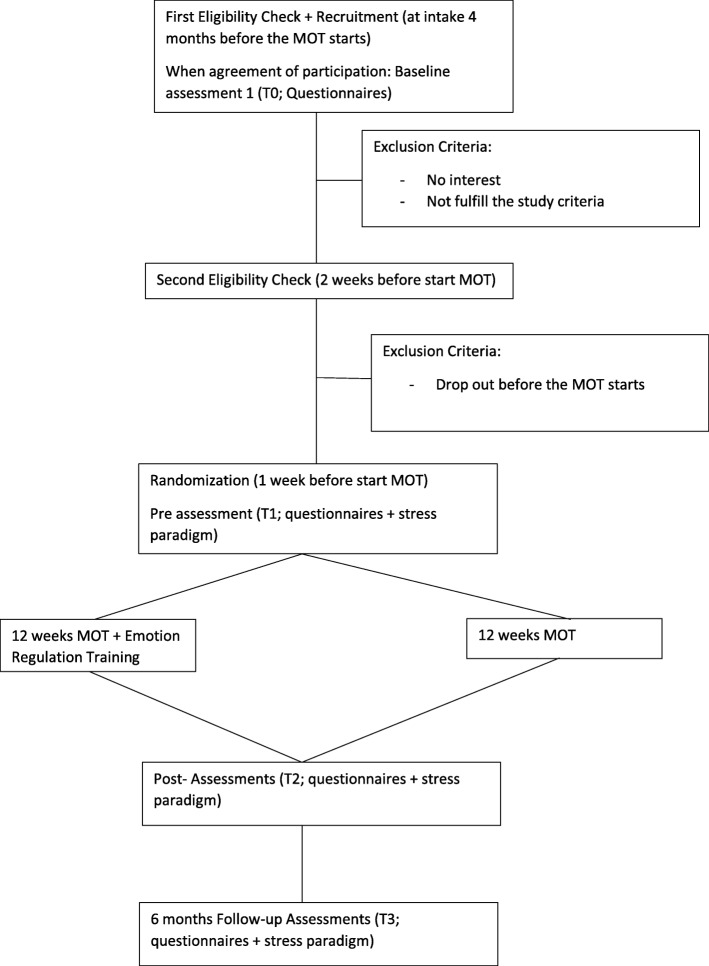
Process of eligibility, allocation, and assessments

**Fig. 3:**
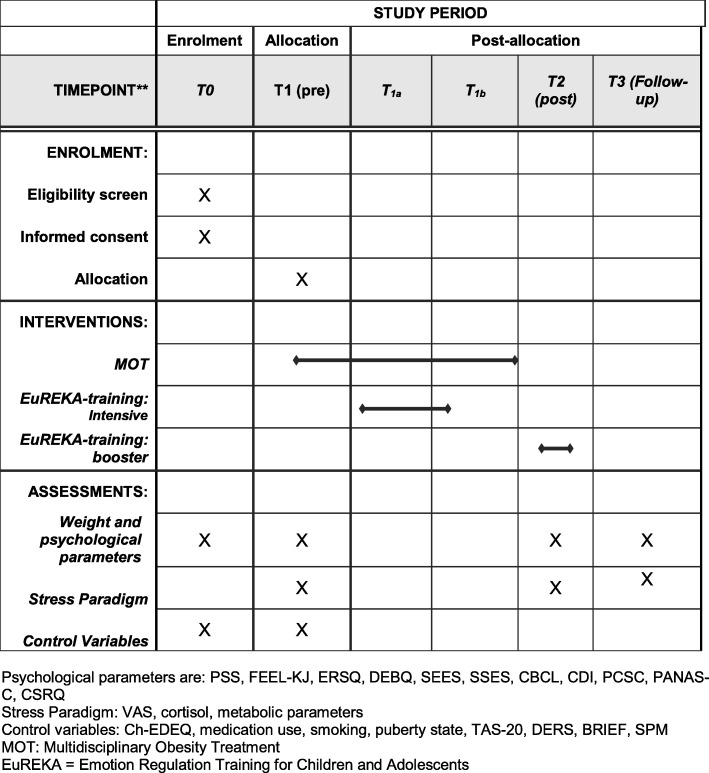
Overview of enrolment, allocation, interventions, and assessments

The eligibility, allocation, and assessments are illustrated in Figs. 2 and 3.

### Randomization

Stratified randomization is a two-stage process, used to control for the influence of covariates (baseline characteristics), in the randomization process [135]. In the current RCT study, both sex and age are identified as potential and important covariates. Therefore, separate blocks for each combination of the covariates will be generated (e.g., girls 10–12, boys 10–12, girls 13–14, and boys 13–14).

Participants who give consent to participate the study will be assigned to one of the four blocks.

Afterward, simple randomization to one of two conditions (e.g., experimental vs. care as usual condition) will be applied within each block at the individual level using dice [82]. The dice is thrown by the same researcher in all consecutive waves. Based on our a priori power calculations that 140 participants have to be included, we will recruit participants until each of the blocks has 35 participants.

### Primary outcome measures

#### Stress paradigm

**Fig. 4:**
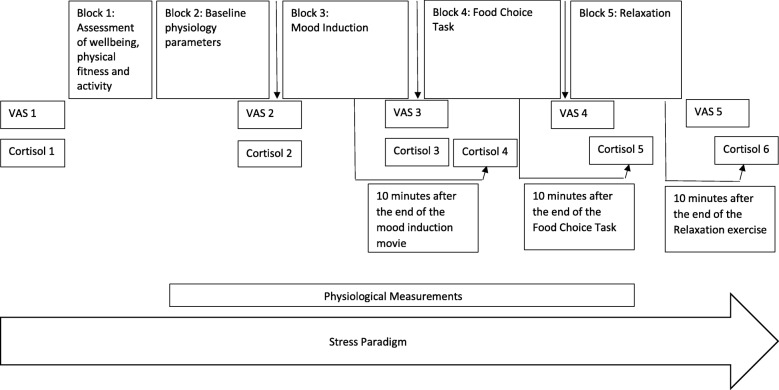
Experimental paradigm

A lab task was developed for assessing stress and emotion regulation on different levels (e.g., cortisol and physiological parameters, respectively), consisting of five blocks as follows. (1) Hunger, physical fitness, and activity of the last 24 h (visual analog scales (VAS), as a confounder for the physiological parameters) will be assessed. (2) A 16-channel portable system for physiological research (Porti 16-channel-amplifier; TMSi, Twente Medical Systems International, EJ Oldenzaal, The Netherlands) will be attached, and 16-min baseline physiology parameters (i.e., 8 min in a neutral condition (looking at a white cross on a black computer screen) and 8 min in a vanilla neutral condition) will be measured. In the latter, the WALL-E movie (T1) and two different 8-min parts of the Denali movie will be used (T2 and T3). (3) Mood induction will occur via validated fragments of “The Champ” (T1), “The Lion King” (T2), and “Father and Daughter” (T3) [47, 126]. Different baseline and mood induction movies will be used at the different measurement points to prevent a habituation of the participants to the film fragments and to be able to repeatedly establish a negative mood. (4) Then, a food choice task (Leeds Food Preference Questionnaire task, LFPQ task) [52] and (5) a relaxation exercise will be undertaken. VAS will be scored to rate the stress and affect level and cortisol samples will be collected to validate the stress self-reports, at different time points during the stress paradigm (see Fig. 4). All the above-mentioned measurements (e.g., VAS, physiology parameters, cortisol, and the LFPQ task) will be discussed below.

#### Stress

##### Visual Analog Scales (VAS)

During the stress paradigm, the participants will rate their stress and affect level at five different time points (see Fig. 4) on a 100-mm line. The affects participants will rate are “bored,” “anxious,” “frustrated,” “sad,” “happy,” “stressed,” and “angry.” Different scores between the VAS on different time points for different affects will indicate changes in mood.

##### Cortisol

To measure stress during the stress paradigm, salivary cortisol will be collected at six time points (see Fig. 4). Salivette synthetic swabs (Sarstedt, Germany), specifically designed for salivary cortisol analysis, will be used. The swabs will be centrifuged for 4 min at 3000 rpm (Jouan CR412 centrifuge), and the filtrates will be stored at − 80 °C [100, 143]. The routine laboratory of the Ghent University Hospital will assay the salivary cortisol by means of a Modular E 170 immunoanalyzer system (Roche Diagnostics, Mannheim, Germany) using the Roche Cobas Cortisol assay. For a more detailed description of this analysis technique, *see* van Aken, Romijn, Miltenburg, and Lentjes [139]. Next, the cortisol concentrations from the analysis (μg/dL) will be multiplied with a conversion factor of 27.586, resulting in the SI units nanomoles per liter (nmol/L) [41]. The difference in the cortisol level throughout the five blocks (stress paradigm) will represent the adaptation or maladaptation to the stress and thus act as a proxy of eustress and distress (*see* Fig. 1).

#### Emotion regulation

##### Fragebogen Zur Erhebung der Emotionsregulation Bei Kindern und Jugendlichen (FEEL-KJ)

The self- and parent report of the Dutch version of the Fragebogen Zur Erhebung der Emotionsregulation Bei Kindern und Jugendlichen (FEEL-KJ) [25, 66] assesses emotion regulation competencies in children and adolescents between 8 and 18 years old. The total amount of items is 90, divided in 15 ER competencies, all measured for three emotions: anger, anxiety, and sadness. The 15 ER competencies are divided in three categories: adaptive (e.g., cognitive reappraisal), maladaptive (e.g., rumination), and external regulation (e.g., social support). Items are answered on a five-point Likert scale (from 0 = not at all to 4 = almost always). For the Dutch and Flemish population, representative norms are available [25], and good reliability and validity were found [40].

##### Emotion Regulation Skill Questionnaire (ERSQ)

The Dutch version of the Emotion Regulation Skill Questionnaire (ERSQ) [14, 19] consists of 27 items and assesses nine different ER abilities and strategies, each operationalized in three items: (1) awareness, (2) (physical) sensations, (3) clarity on which emotions were experienced, (4) understanding of why an emotion occurs, (5) modification/influencing the emotion, (6) acceptance of the emotion, (7) tolerance of the emotion, (8) readiness to confront situations and (9) self-support. Items are answered on a five-point Likert scale (from 0 = not at all to 4 = almost always). Nine subscale scores and one total score for successful ER abilities and strategies can be assessed [62]. Good psychometric qualities were found for the ERSQ, and the questionnaire has been evaluated as reliable and valid [19, 62].

##### Idiosyncratic measures

Participants in the intervention condition (MOT + ER training) will be asked to fill out a diary in the 3 days between the ER training sessions. The diary consists of VAS (from 0 to 100) to assess the amount of stress, negative affect (anxiety, sadness, anger) and positive affect (happy) and five-point Likert scales (from 1 = completely not true to 5 = completely true) to assess the use of the ER ability “emotional awareness” and the ER strategies “distraction, cognitive reappraisal and problem solving” for the three measured negative feelings.

##### Respiratory sinus arrhythmia (RSA)

RSA, a physiological indicator of the parasympathetic nervous system, is shown to be an objective assessment of emotional responding and regulation [12, 117, 118, 128]. RSA is determined by the heart rate acceleration and deceleration during inhalation and exhalation, respectively [13, 117, 118], and reflects the parasympathetic influence on heart rate variability (HRV, defined as the variation over time of the period between successive heartbeats) [1, 20]. Numerous studies have linked low levels of resting RSA (at baseline) and large reductions in RSA reactivity (after exposure to a high emotionally eliciting stimulus) to psychiatric disorders characterized by emotion dysregulation, such as depression and anxiety [12, 128]. These findings have been confirmed in children, adolescents, and adults [12, 33, 140, 145].

The autonomic responses, more specifically electrocardiogram (ECG), will be recorded, by means of a 16-channel portable system for physiological research (Porti 16-channel-amplifier; TMSi, Twente Medical Systems International, EJ Oldenzaal, The Netherlands) and the software Polybench 1.2 (TMSi) to measure the RSA. The signals will be digitized with a 1000 Hz-sampling rate and will be processed in ANSLAB software, a computer program written in MATLAB [22]. R-waves will be detected automatically, followed by a visual check and the edit of artifacts. For assessment of the ECG, throwaway Ag/AgCL solid-gel electrodes with a diameter of 55 mm will be attached to two skin sites: at the right upper sternum and under the left lowest rib. Additionally, a ground electrode will be fixed on a ground wristband on the dorsum of the nondominant wrist. As shown in Fig. 4, the physiological measures will be recorded between the start of block 2 and the start of block 5.

Resting RSA will be recorded during 16-min baseline (block 2, *see* Fig. 4). In both the neutral and vanilla neutral baseline condition, only the final 5 min of the ECG recording will be used for further analyses to ensure that the participants are habituated to the laboratory setting, which increases reliability [43]. RSA reactivity will be quantified as the RSA measures during the mood induction video clip (block 3, see Fig. 4) and the food choice task (block 4, see Fig. 4) minus the baseline RSA [12, 140].

#### Emotional eating

##### Dutch Eating Behavior Questionnaire (DEBQ)

The self-report and parent report of the Dutch Eating Behavior Questionnaire (DEBQ) [24, 141] assess three eating styles: restrained, external, and emotional eating. In this study, only the subscale “emotional eating” will be considered. In total, the DEBQ contains 33 items, of which 11 items belong to the subscale “emotional eating,” rated on a five-point Likert scale (from 1 = never to 5 = very often). For both the DEBQ total and the subscale “emotional eating,” good reliability and validity have been reported, and the questionnaire is shown to be useful in research with children and adolescents [24, 26, 121].

##### Salzburg Emotional Eating Scale (SEES) and the Salzburg Stress Eating Scale (SSES)

The Salzburg Emotional Eating Scale (SEES) and the Salzburg Stress Eating Scale (SSES) are developed to measure emotional and stress-related under- and overeating [98, 99]. The self-report questionnaires contain 20 and 10 items, respectively. The items are formulated as emotional and stressful events of which the participant needs to complement the sentence with one of the five-point Likert scale answer alternatives: 1 = I eat much less, 2 = I eat less, 3 = I eat just as much, 4 = I eat more, and 5 = I eat much more. The SEES questionnaire contains a four factor structure with the following four subscales: happiness, sadness, anger, and anxiety. For each subscale a good reliability, internal consistency and validity was found [98]. The SSES questionnaire has a one-factor structure and is found to have good internal consistency [99].

##### Idiosyncratic measures

Three items are added to the diary (see idiosyncratic measures above) to measure the effect of eating on the current mood (e.g., “When I felt bad today, and I would eat something, I would feel less bummed out and miserable”). Participants will answer the items on a five-point Likert scale (from 1 = Strongly disagree to 5 = Strongly agree). The items are based on the Trait and State General Food- Cravings Questionnaire (G-FCQ-T, G-FCQ-S) [108].

##### Leeds Food Preference Questionnaire task (LFPQ)

The Leeds Food Preference Questionnaire (LFPQ) task [54] is a computer-based paradigm designed to assess explicit wanting and liking of food, and implicit wanting of food. The stimuli are 20 food images varying in fat content (high or low) and taste properties (savory or sweet). Based on these dimensions, four categories of food can be separated (high fat savory, low fat savory, high fat sweet and low fat sweet) including each five food stimuli [52–54]. In the explicit task, the 20 food items are shown consecutively on the computer screen, with the following two questions: (1) “How pleasant would it be to consume this food at this moment?” and (2) “How much do you want some of this food right now?” The participants rate the items on 100 mm VAS ranging from “Not at all” to “Extremely”, by moving the computer mouse. The implicit wanting task consists of a “forced choice methodology”. During this implicit task, two food items of different food categories are shown on the screen. In total, 150 trials are presented with the same standardized instruction: “*choose the food item you want to eat most right now*”. In this implicit wanting task, two outcome measures are registered: the frequency of selected food items in each category (range = 0–75; reflecting a relative preference) and reaction times (milliseconds) of every choice [52–54]. Good psychometric qualities are found and the LFPQ task is widely used in different contexts [35].

### Secondary outcome measures

#### Weight index and related metabolic parameters

##### Adjusted Body Mass Index (AdjBMI)

The Adjusted Body Mass Index (AdjBMI) will be used to objectify the weight index of the participants. This means that the BMI will be calculated and based on normative Flemish data [122] expressed as percentage above the mean, according to age and gender. Cutoff criteria for childhood overweight and obesity, based on age and gender growth charts, are proposed by the International Obesity Task Force (IOTF). These criteria are 25 kg/m^2^ and 30 kg/m^2^, respectively [38].

##### Waist circumference and waist circumference-to-height

*Waist circumference* will be measured to 0.1 cm at the level of the iliac crest [30, 81]. Percentile reference charts for waist circumference have not yet been developed on a European and Belgian level [105]. Therefore, z-scores will be used to make the variable age- and gender-independent. *Waist circumference-to-height* (ratio waist circumference on height) will be computed, as recent findings suggest that it predicts adiposity better than BMI and waist circumference [30]. Moreover, a cutoff score for waist circumference-to-height is set on 0.5, related to increased health risks in children [93].

##### Biological samples

To later validate or explain some of the observed intervention effects, several biological samples will be collected, e.g., for inflammation analysis. A passive drool saliva sample is taken at the start of the stress paradigm (T1, T2, T3). In a subsample, fasting venous blood and stool samples are collected (T1, T2).

#### Psychological well-being

##### Child Behavior Check List (CBCL) and Youth Self Report (YSR)

The Child Behavior Check List (parent version) and Youth Self Report (child version) assess emotional and behavioral problems in children and adolescents between 6 and 18 years old (CBCL) and 11 and 18 years old (YSR) [2]. Both questionnaires contain 118 items that need to be answered on a three-point Likert scale (0 = never, 1 = sometimes, and 2 = often). Three subscale scores can be computed for both questionnaires: total, internalizing, and externalizing problems. Good reliability and validity have been found for both questionnaires [2].

##### Children’s Depression Inventory (CDI)

The Children’s Depression Inventory (CDI) [85, 137] contains 27 items and assesses depressive symptoms in children and adolescents (7–17 years old). Participants need to read three sentences and have to choose the sentence that best describes them during the previous 2 weeks. Scores assigned to the sentences are 0, 1, or 2, with higher scores indicating more depressive symptoms. Much empirical research has strongly confirmed the good reliability and validity of the questionnaire [85].

##### Perceived Competence Scale for Children (PCSC)

The child (8–12 years) and adolescent (12–18 years) Dutch version of the Perceived Competence Scale for Children (PCSC) [72, 138] assesses the self-perception of competence feelings, both positive and negative. The child version of the PCSC consists of four subscales measured by 28 items: (1) cognitive abilities, (2) physical appearance and activity, (3) social competence, and (4) general self-worth. The adolescent version of the PCSC contains 35 items and includes three additional subscales: (1) sporting competence, (2) close friendships, and (3) overall attitude. Acceptable to good test-retest reliability, good internal reliabilities, and factor validity have been reported [61, 72].

##### Positive and Negative Affect Scale for children (PANAS-C)

The Positive and Negative Affect Scale for children (PANAS-C) [87] measures two trait temperament dimensions, Negative Affect and Positive Affect. Both subscales contain 15 items, existing out of 15 emotions that need to be rated on a five-point Likert scale (from 1 = “very slightly” to 5 = “very much”). Good psychometric qualities have been reported for this questionnaire [87].

#### Physical well-being: sleep quality

##### Chronic Sleep Reduction Questionnaire (CSRQ)

The Chronic Sleep Reduction Questionnaire (CSRQ) [46] consists of 20 items to measure sleep reduction, with four subscales: (1) shortage of sleep, (2) irritation, (3) loss of energy, and (4) sleepiness. The items are answered on a three-point Likert scale (with 1 = no, 2 = sometimes, and 3 = yes). Good psychometric qualities have been reported for the CSRQ. For the four subscales, good validity and acceptable to good reliability have been found [46].

### Control variables

We will control for multiple variables that are known as important but without specific hypothesis regarding their effect on our outcome variables, e.g., eating disorders, medication use, smoking behavior, puberty state, alexithymia, and cognitive functioning. First, eating problems, such as loss of control and binge eating are often observed as comorbidities of obesity [59, 152], associated with more eating-related and internalizing psychopathology [59]. Second, medication use is associated with a lower heart rate variability [112]. Third, smoking behavior can be a confounder in the relation between stress and weight, as it is a maladaptive ER strategy. Moreover, smoking can influence the energy imbalance [154]. Fourth, obesity is associated with early puberty onset, initiated by high levels of leptin [131]. Besides, the pubertal development influences the stress regulation by changes in the cortisol axis [94]. Fifth, alexithymia is a construct referring to difficulties in identifying and describing emotions [136]. In addition, alexithymia is associated with difficulties in discriminating between different emotions [10] and with difficulties in coping with stressful events [115]. Sixth, cognitive functioning, more specifically executive functioning, is shown to play a role in the development and maintenance of overweight and obesity [57, 65].

#### Eating disorders: Children’s Eating Disorder Examination-Questionnaire (Ch-EDEQ)

The Dutch translation of the Children’s Eating Disorder Examination-Questionnaire is a self-report questionnaire (CH-EDEQ) (Decaluwé and Braet: Dutch translation of the child eating disorder examniation, unpublished) [49, 50] based on the Children’s Eating Disorder Examination (Ch-EDE) [51]. The Ch-EDEQ can be used in children and adolescents from the age of 8 years, and it consists of 22 items. The scale assesses three types of eating behavior: (1) objective overeating, (2) objective binge eating, and (3) subjective binge eating, and it contains four subscales questioning eating disorder psychopathology: (1) restraint eating, (2) eating concern, (3) shape concern, and (4) weight concern. Participants need to indicate how many days during the last month the behavior occurred. Good psychometric characteristics have been reported [45].

#### Medication use

Medication use will be questioned by one item: “Do you use medication?” with two answer alternatives: “yes” or “no.” When the participant answers “yes,” he or she will be asked to specify the type and amount of medication.

#### Smoking

Smoking will be questioned by one item: “Do you smoke?” with two answer alternatives: “yes” or “no.” When the participant answers “yes,” he or she will be asked to specify the amount of cigarettes a day.

#### Puberty state – Tanner’s stage

The puberty state and sexual maturation will be measured by two self-report questions on hair and genital development. The participants need to choose between five drawings of genitals (corresponding with the five pubertal stages) and need to indicate the drawing that is most associated with his/her own genital development. Good psychometric characteristics have been reported [130].

#### Alexithymia: Toronto Alexithymia Scale-II (TAS-20)

The Toronto Alexithymia Scale-II measures alexithymia with 20 items (TAS-20) [11]. The items are scored on a three-point Likert scale (from 1 = not correct for me to 3 = correct for me). The questionnaire contains three underlying correlated factors: (1) difficulties in identifying emotions, (2) difficulties in describing emotions to others, and (3) an externally oriented thinking style [114]. Good internal consistency and validity have been shown in previous research [11].

#### Alexithymia: Difficulties in Emotion Regulation Scale (DERS)

The Difficulties in Emotion Regulation Scale (DERS) [63] assesses six possible difficulties in ER: (1) lack of awareness of emotions, (2) lack of clarity of emotions, (3) nonacceptance of emotions, (4) limited access to ER strategies, (5) difficulties controlling impulsive behavior when having a negative feeling, and (6) difficulties in goal-directed behavior when experiencing a negative feeling. In this study, only the first dimension will be questioned by six items. The participants need to answer on a five-point Likert scale (from 1 = never to 5 = almost always). High internal consistency, good test-retest reliability, and good validity have been found [63].

#### Cognitive functioning: Behavior Rating Inventory of Executive Functioning (BRIEF)

Executive functioning will be measured by means of the Behavior Rating Inventory of Executive Functioning (BRIEF) parent-report [77]. The parent-report version contains 75 items, divided in seven subscales: (1) inhibition, (2) cognitive flexibility, (3) emotional regulation, (4) initiation, (5) working memory, (6) planning/organizing, and (7) monitoring. Respondents need to answer on a three-point Likert scale (0 = never, 1 = sometimes, and 2 = often). Good psychometric characteristics have been reported [77].

#### Cognitive functioning: Standard Progressive Matrices test (SPM)

The Standard Progressive Matrices test will be included to measure the general cognitive ability of the participant. Five blocks of items are shown to the participant. The items are different figures following a certain logic. The participants need to choose the correct figure in a list of six or eight answer alternatives to complete the logic. Each sets starts with easy items which become more difficult throughout the set [120].

### Interventions

#### Inpatient treatment at the Zeepreventorium VZW (MOT)

All participants, both in the intervention and control condition, will receive an inpatient Multidisciplinary Obesity Treatment (MOT) during 12 months, starting from July. The primary aim of the MOT is to obtain a healthy body weight by three main therapeutic components: daily physical activity, healthy diet, and cognitive behavioral therapy. Also, the parents are involved in the treatment (e.g., via psycho-educational moments) [29].

#### Emotion regulation training

The emotion regulation training is called “EuREKA,” an acronym for “ a Dutch translation of ‘Emotion Regulation training for Children and Adolescents.” The content and structure of the EuREKA-training will be shortly discussed below. Both a manual and a workbook are available.

##### Theory

**Fig. 5:**
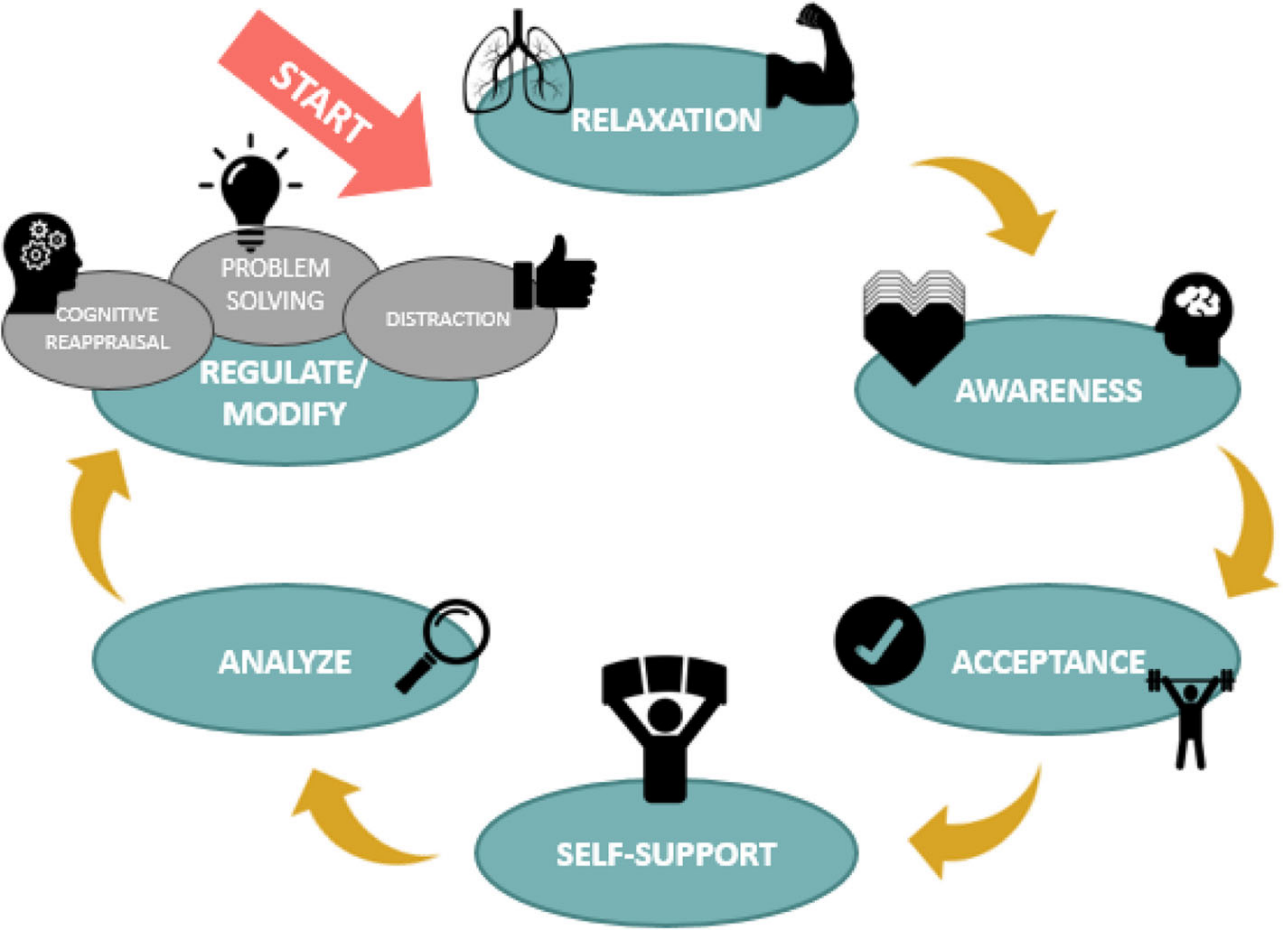
EuREKA- sequence

As mentioned in the introduction, Berking and Whitley [18] developed an Affect Regulation Training (ART) for adults, which is well-evaluated in both clinical and nonclinical samples [15, 17, 18, 60]. Based on this ART, EuREKA has been developed for use in younger age groups, with several adjustments: an adjustment of terms (e.g., simplified wordings for the ER sequence, *see* Fig. 5), child-friendly adapted psycho-education, and exercises evaluated as feasible for children and adolescents.

##### Content

All ER competences will be learned to the participants by means of (1) psycho-education and (2) exercises, both adjusted to the population of children and adolescents. For example, the psycho-education will be given by movie clips. Also, the exercises related to the different competences have been evaluated already in schoolchildren [147] and tested in children with obesity. After every EuREKA session, the participants will receive homework to exercise the learned competence.

##### Structure

The EuREKA training consists of 12 sessions, one session of 2 hours per week. Participants will be divided in groups of five youngsters, based on their age. The choice for homogenous groups, based on age, has the following reasons: (1) the adjustment of the language during psycho-education and exercises is easier when all children have the same age, and (2) the examples can be more life phase oriented. During every EuREKA session, two trainers will be present: one trainer will focus on the content of the program, while the second trainer will monitor the process in the group (e.g., group dynamics, time management). Every EuREKA session is organized in the same structure to promote predictability and safety. First, every session starts with a short recapitulation of the previous session in which the learned competence and the accompanying homework are discussed. Second, the new competence is introduced and placed into the sequence, positioned on the previous learned competences. Third, the new competence is learned in depth, which is the biggest part of the training session, with psycho-education and exercises on the new skill. Fourth, all learned competences until that session are exercised in the sequence by an imagination exercise. Fifth, the homework of that specific session is explained, and the participants receive a little incentive for their cooperation and motivation during the session (see below, motivational processes).

#### Drop-out, feasibility and motivational processes

Unfortunately, drop-out rates are quite high in treatment of obesity [132]. Therefore, increasing the motivation is important at the start of treatment, which will be emphasized during every EuREKA session. Moreover, to raise the motivation in the current study, participants in both the intervention and control group will receive a cinema voucher after completing the stress paradigm before (T1) and after the intervention (T2). Additionally, after every EuREKA session, the children in the intervention group will receive a little incentive, adjusted to the content of that particular EuREKA session. To test the feasibility of the ER training, after every session, the trainers and the participants will score the different components of the training on VAS (0–100 mm). Both the content of the training and the features of the session (e.g., group safety, attitude of the trainer) will be assessed. These insights aim to help understanding the drop-out and to improve the adherence rates [133]. Moreover, also the treatment integrity of the EuREKA training will be evaluated. Therefore, all sessions will be recorded and scored on VAS (0–100 mm) by independent raters.

### Data analysis

#### Sample size

To detect a clinically relevant treatment effect in both primary and secondary treatment outcomes, the power analysis was conducted on the AdjBMI index [80]. To detect a minimally important difference in the in AdjBMI-index (− 0.5 BMI points), a power analysis showed that 63 participants in every condition are necessary to obtain a power of 80% at a significance level of .05. As drop-out rates are high in inpatient treatment centers [132], we will include 10% more participants. This calculation leads to the inclusion of 70 participants in both the intervention and control condition.

#### Data storage

A research data management plan has been formulated, in which the collected data and data storage have been defined. The plan defines the location on the computer where the data is stored and who is responsible for the data.

#### Analytical plan

To solve the problems of noncompliance and missing outcomes, which are iterative complications in an RCT study, the intention-to-treat (ITT) principle will be used [69]. Multilevel analyses will be used to test the treatment effects, allowing handling of the missing and nested data from the two conditions (intervention versus control condition). More specifically, mixed models with fixed effects for condition (intervention versus control condition), time (immediately after intervention and 6 months’ follow-up), and their interaction, adjusted for baseline will be used.

## Discussion

The current RCT study emphasizes the growing problem of obesity, which is bidirectionally associated with the occurrence of stress (see Fig. 1) by three processes: (1) cortisol hypersecretion, (2) low-grade inflammation, and (3) emotional eating. A lack of emotion regulation competencies is hypothesized as an important intermediate factor. Therefore, the current RCT study aims to examine the effectiveness of ER Training on top of a Multidisciplinary Obesity Treatment (care as usual) in inpatient-treated youngsters (10–14 years old). The ER Training “EuREKA” is an innovative intervention program for children and adolescents, based on the evidence-based ART for adults developed by Berking and Whitley [18]. Previously, in this age group, we first tested the program in schools and designed a feasibility study in obese youngsters [147]. We hypothesize that EuREKA in addition to the MOT will result in 1) an improvement in ER competencies, more specifically having more adaptive emotion regulation strategies; 2) less emotional eating behavior; 3) better sleep quality; 4) improved weight-loss and weight-loss maintenance; and 5) better long-term (6-month) psychological well-being.

Results of this RCT study will add to the scientific and clinical literature on the role of ER in the development and maintenance of pediatric obesity. These results will be important as the role of ER in the stress-obesity relationship will be explored and elucidated. Moreover, as ER is a transdiagnostic factor and thus of importance in many psychopathologies, this study will add relevant information to the broad psychology literature. An additional important contribution of the current RCT study will be the new therapeutic insights to improve the long-term effects of existing obesity treatments [4, 96]. If these first RCT results are promising, future research should replicate these effects and can set the stage for offering the training in a new format (e.g., a digital m-health tool).

A first strength of the study is the use of a rigorous design, including a care-as-usual control group (MOT), randomization of the participants and six-month follow-up measurements to evaluate the effect of the EuREKA-training in an inpatient treatment for obesity [56]. A second strength is the use of different measurement methods (questionnaires, stress paradigm, and physiological measures) and informants (youngsters and parents) at all four data collection time points (T0, T1, T2, and T3). A third strength is the well-evaluated ART model and training, which has been the basis for the development of the EuREKA training that will be used in this study [16, 18]. Fourth, including homework in the EuREKA training is an advantage since it has the potential to increase the feeling of competence of the learned competencies in the training sessions [76].

This study also has some limitations. First, the control group is passive, and we will be unable to control for several aspects such as extra attention, rewards, and homework in the intervention group in comparison with the control group. Second, practical obstacles are expected. Therefore, a feasibility study preceding the RCT study in an inpatient treatment center for obesity was conducted, and practical difficulties concerning the organization, such as planning the extra therapy during school hours, were taken into account. However, new unforeseen obstacles cannot always be avoided. Second, no double-blind paradigm can be used, as the researchers will know who belong the EuREKA training intervention group. A consequence is a possible contamination in the instructions by the researchers and the participants of the intervention condition trying to perform better (Hawthorne effect) [78]. However, this lack is common in educational research trials [78].

## Conclusion

The current RCT study will evaluate an innovative emotion regulation intervention program, EuREKA, in obese children and youngsters (10–14 years) in an inpatient treatment program. When the EuREKA training is found to be effective, causal proof of ER’s role in overweight and such ER intervention can be applied in clinical practice, e.g., after translation into a digital m-health tool.

## Trial status

The recruitment started in March 2018 and will end in September 2019. The RCT study protocol was registered in the ISRCTN Registry with study ID “ISRCTN 83822934” on 13 December 2017 (http://www.isrctn.com/ISRCTN83822934).

## Supplementary information

**Additional file 1.** Informed consent.

## Abbreviations

ER: Emotion regulation
EuREKA: Emotie Regulatie Training voor kinderen en adolescenten = Emotion Regulation Training for Children and Adolescents
LFPQ: Leeds Food Preference Questionnaire task
MOT: Multidisciplinary obesity treatment
