# Supplementary material for: Emotion regulation training in the treatment of obesity in young adolescents: protocol for a randomized controlled trial
Source: Trials. 2020 Feb 10;21:153. doi: 10.1186/s13063-019-4020-1 (PMC7011608; doi:10.1186/s13063-019-4020-1)
Supplement: Supplementary file 1 — Additional file 1. Informed consent. [file 13063_2019_4020_MOESM1_ESM.pdf]

**P3-6: Informed consent parents**

(A) Goal of the study

Stress and negative affect are playing an important role in the development of food preferences, overweight and obesity. In the current study we want to train youngsters with obesity to cope with their stress and negative emotions. We want to study if this training is helpful to develop an adaptive emotion regulation; and if the training is helpful in obtaining and maintaining a healthy weight.

(B) Progress of the study

1. After giving an informed consent, your son/ daughter will possibly participate in an emotion regulation training (not when the youngster is in the control condition)
2. Your son/daughter will participate in a 12 weekly training (2 hours a week) to train emotion regulation strategies: learning to cope in a good way with stress and negative emotions. In the training, movie clips, exercises, games ,… will be used. The training will be given in little groups of 4 until 5 youngsters. The training will be given during the stay of your son/daughter in the residential setting.
3. To examine if the training has extra advantages in comparison with the current treatment, we will ask the youngsters
   a) To complete some questionnaires, before the start of the training, at the end of the training and at 6 months after the training.
   b) To complete a diary in between the training sessions about their feelings and the way your son/daughter is coping with these feelings.
   c) To participate a labtest in which we will show the youngsters a stressful movie. We want to examine how the youngsters cope with this movie clip. After the movie clip, the youngsters will do a computer task to examine their food preferences; and the heart rate will also be measured during the task. This will be done before the training, at the end of the training and at 6 months follow-up.
   d) To collect saliva samples during the lab task. A subsample of the youngsters will be asked to collect 2 times blood and stool samples. With these samples, we want to look to the effect of stress, and to the effect of the training.

4. If you wish, we can send you the results of the measurements, after completion of the research.

<u>Approval of the parents: informed consent:</u>

We want to ask you to sign the informed consent when you and your child want to participate the study.

“it is clear to us that the participation to the study is completely voluntary and we can stop the study at each moment, without giving a reason. Not participate the study or redraw out of the study will not be disadvantageous for us(/our) child(ren). We did receive the information on the study, we had the chance to ask questions about the study, we know who to contact if we have questions in the future.”

**P7: Informed consent children and adolescents**

(A) Goal of the study

We want to investigate if coping differently with your negative feelings and stress can help in the treatment of obesity. Therefore, we want to present a nice training of one session a week (2 hours) during 12 weeks. In little groups of 4 until 5 youngsters, we will talk about feelings, how to cope with your feelings, playing games, looking to movie clips,… We think you will be able to cope better with your stress and negative emotions, you will be able to lose weight more easily and you will be able to better maintain your healthy weight.

(B) What is expected from you?

1) To participate a training during your stay in Het Zeepreventorium
2) To complete some questionnaires and a diary
3) To participate a lab task three times during your stay in Het Zeepreventorium to measure your heart rate, collect saliva, do a computer task on food preferences and look to a movie.
4) (Maybe, only if you want to): collect 2 times blood and feces.

(C) What do you need to know?

Only you can decide if you want to participate the study. Participating or not participating has no consequences for you or your parents. Your personal information and answers will be processed and published anonymously, and will not be shared with others.

You can stop participating the study at every moment.

You can ask for more information at every moment.

# RCT- STUDIE: STRESS BIJ OBESITAS OUDERS

## INFORMATIE OMTRENT HET ONDERZOEK

### (A) DOEL VAN DE STUDIE

Er bestaan sterke aanwijzingen dat stress en negatieve emoties een rol spelen in de ontwikkeling van voedingsvoorkeuren, overgewicht en obesitas. In het huidig onderzoek willen we jongeren met obesitas een training aanbieden die hen leert om op een helpende manier om te gaan met stress en negatieve emoties. We willen nagaan of deze training inderdaad helpend is voor een goede emotieregulatie én voor het bekomen en behouden van een gezond gewicht.

### (B) VERLOOP VAN HET ONDERZOEK

1. Nadat u akkoord gaat, zal u zoon/dochter de emotieregulatie training mogen meevolgen in het Zeepreventorium.
2. Uw zoon/dochter zal gedurende 12 weken een training kunnen volgen (2uur per week) om op een goede manier met stress en negatieve gevoelens te leren omgaan. In deze training zal gewerkt worden met filmpjes, spelletjes, oefeningen, ... De training wordt in groepjes van 4 of 5 jongeren gegeven. De training zal volledig verlopen tijdens het verblijf van uw zoon/dochter in het zeepreventorium.
3. Om te onderzoeken of deze training extra voordelen oplevert tegenover het enkel volgen van de huidige therapie, zullen we vragen aan de jongeren
    a. enkele vragenlijsten in te vullen, voor de start van de training, na het einde van de training en 6 maand na het einde van de training.
    b. tussen de trainingssessies in een dagboekje in te vullen omtrent de stemming en de manier waarop uw zoon/dochter omgaat met gevoelens.
    c. deel te nemen aan een proefje waarin we een stresserend filmpje laten zien. We willen nagaan hoe de jongeren hiermee omgaan. Hierbij zal ook een smaaktest gebeuren en zal de hartslag van uw kind gemeten worden. Dit zal plaatsvinden voor de start van de training; na het einde van de training, en 6 maand na het einde van de training.
    d. tweemaal een bloedstaal en een stoelgangstaal (via een meegegeven opvangbakje in het toilet) af te staan namelijk voor en na de training. Hiermee willen we kijken naar het effect van de stress, en het leren van het omgaan met deze stress op een aantal biologische parameters die van invloed zijn op de gewichtscontrole.

4. Indien u dit wenst, kunnen we u na afloop van dit onderzoek de resultaten van bepaalde metingen opsturen, zoals bv. vragenlijstresultaten omtrent het welzijn van uw kind, bloedparameters van suiker en ontsteking en lichaamssamenstelling.

## (C) VEREISTEN VAN DEELNAME

- De jongere is bereid om deel te nemen aan een emotieregulatie training, tijdens het verblijf in het zeepreventorium, vragenlijsten en een dagboekje in te vullen, 2 maal deel te nemen aan een proefje om de stressreacties te observeren en een stoelgangstaal te verzamelen.
- De ouder is bereid om een dagboekje in te vullen tijdens de weekends als de jongere thuis is.

## (D) RISICO'S BIJ DEELNAME

Er zijn geen risico's of bijwerkingen verbonden met het onderzoek.

## (E) WETTELIJK KADER

Deze studie zal volledig conform de voorschriften van de Europese en lokale wetgeving rond Clinical Research, in het bijzonder ook de Belgische wet van 7 mei 2004 inzake experimenten op de menselijke persoon, verlopen en respecteert ook alle bijkomende regelingen betreffende de bescherming van individuen, betrokken in medisch onderzoek. Alle gegevens die we tijdens de studie verzamelen zullen strikt vertrouwelijk behandeld worden, conform de Belgische wet van 8 december 1992. De studie werd goedgekeurd door het Onafhankelijk Ethisch Comité van het UZ Gent. In overeenstemming met de Europese Wetten en conform de Belgische wet van 7 mei 2004, heeft de Universiteit Gent een verzekeringsbeleid afgesloten teneinde onvoorziene gebeurtenissen tijdens de studie te dekken. U dient deze goedkeuring echter niet te beschouwen als een aanzet tot deelname aan de studie, u kan op elk moment de studie stop zetten.

## (F) CONTACT

Bij vragen of problemen kan u altijd terecht bij:

Taaike Debeuf
Telefoon: 09 264 64 21
Email: Taaike.Debeuf@ugent.be
Adres: Henri Dunantlaan 2, 9000 Gent

Dr. Sandra Verbeken

Telefoon: 09 264 64 21
Email: Sandra.Verbeken@ugent.be
Adres: Henri Dunantlaan 2, 9000 Gent
Onderzoek onder leiding van Prof. Dr. Caroline Braet en prof. Dr. Stefaan De Henauw.

## TOESTEMMING DOOR OUDERS / INFORMED CONSENT

We willen vragen om deze toestemmingsverklaring te tekenen indien u en uw kind willen deelnemen aan deze studie.

Het is ons duidelijk dat onze deelname en die van ons(/onze) kind(eren) compleet vrijwillig is en dat we ons op elk moment uit de studie kunnen terugtrekken zonder hiervoor een reden op te geven. Niet deelnemen of ons terugtrekken uit de studie zal niet leiden tot nadelen voor ons(/onze) kind(eren). We hebben de informatie verkregen. We hebben de kans gekregen om vragen te stellen over de studie en we weten waar we terecht kunnen als we in de toekomst nog vragen hebben.

Ja ☐ Neen ☐

**HANDTEKENING:**

Ouder 1:______________________________

Ouder 2:______________________________

**Datum:** ……../……../20……

## INFORMATIE EN TOESTEMMINGSVERKLARING DOOR TIENERS

Indien je 12 jaar of ouder bent is het belangrijk dat ook jij toestemming geeft om mee te werken aan het onderzoek. We willen je daarom vragen om dit formulier te lezen en te tekenen als je wilt meewerken.

### (a) DOEL VAN DE STUDIE

We willen onderzoeken of het op een andere manier leren omgaan met gevoelens en stress, kan helpen in het behandelen van obesitas. Om dit te onderzoeken willen we jullie een leuke training aanbieden die bestaat uit 1 sessie van 2uur per week en dit gedurende 12 weken. In kleine groepjes van 4 of 5 jongeren zullen we het hebben over spreken rond gevoelens, hoe je kan omgaan met gevoelens, spelletjes spelen hieromtrent,... We gaan ervanuit dat als je beter met je stress en negatieve gevoelens kan omgaan, je gemakkelijker afvalt en op lange termijn beter je gewicht zal kunnen behouden.

### (B) WAT WORDT ER VAN JOU VERWACHT

1. Dat je tijdens je verblijf in het zeepreventorium deelneemt aan een training waarin je op een andere manier leert omgaan met stress en negatieve gevoelens.
2. Dat je een aantal vragenlijsten invult en een dagboekje bijhoudt.
3. Tijdens de training zal je ook 3 keer naar het lab kunnen komen om jouw hart te laten meten, speekselstaaltjes te nemen, een bloedafname te doen, snackvoorkeur te geven, en naar een film te kijken.
4. Je zal ook gevraagd worden om een staaltje van je stoelgang te verzamelen (kan je helemaal zelf en alleen doen).

### (C) WAT JE MOET WETEN

- Het is jouw eigen keuze mee te doen aan deze studie. Meedoen of niet meedoen heeft geen enkel gevolg voor jou of jouw ouders. Jouw persoonlijke informatie en antwoorden gaan we niet verspreiden naar anderen. Wel worden de resultaten anoniem verwerkt en anoniem gepubliceerd

- Op elk moment kan je stoppen met de studie, ook nadat je hieronder getekend hebt.

- Je kan elk moment meer informatie vragen.

Naam deelnemer: …………………………………………………………………………

Handtekening: Datum: ……/………../20……….
